# A genome-wide association study identifies novel candidate genes for susceptibility to diabetes mellitus in non-obese cats

**DOI:** 10.1371/journal.pone.0259939

**Published:** 2021-12-07

**Authors:** Yaiza Forcada, Mike Boursnell, Brian Catchpole, David B. Church

**Affiliations:** 1 Veterinary Clinical Sciences, The Royal Veterinary College, North Mymms, Hertfordshire, United Kingdom; 2 Canine Genetics, Animal Health Trust, Kentford, Newmarket, Suffolk, United Kingdom; 3 Pathology and Population Sciences, The Royal Veterinary College, North Mymms, Hertfordshire, United Kingdom; University of Lincoln, UNITED KINGDOM

## Abstract

Diabetes mellitus (DM) is a common feline endocrinopathy, which is similar to human type 2 diabetes (T2DM) in terms of its pathophysiology. T2DM occurs due to peripheral insulin resistance and/or β-cell dysfunction. Several studies have identified genetic and environmental factors that contribute to susceptibility to human T2DM. In cats, environmental factors such as obesity and physical inactivity have been linked with DM, although to date, the only genetic association that has been demonstrated is with a polymorphism in the feline *MC4R* gene. The aim of this study was to perform a genome-wide association study (GWAS) to identify polymorphisms associated with feline DM. Illumina Infinium 63k iSelect DNA arrays were used to analyse genomic DNA samples from 200 diabetic domestic shorthair cats and 399 non-diabetic control cats. Data was analysed using PLINK whole genome data analysis toolset. A linear model analysis, EMMAX, was done to test for population structure and HAPLOVIEW was used to identify haplotype blocks surrounding the significant SNPs to assist with candidate gene nomination. A total of 47,497 SNPs were available for analysis. Four SNPs were identified with genome-wide significance: chrA2.4150731 (p_raw_ = 9.94 x10^-8^); chrUn17.115508 (p_raw_ = 6.51 x10^-8^); chrUn17.394136 (p_raw_ = 2.53 x10^-8^); chrUn17.314128 (p_raw_ = 2.53 x10^-8^) as being associated with DM. The first SNP is located within chromosome A2, less than 4kb upstream of the dipeptidyl-peptidase-9 (*DPP9*) gene, a peptidase involved in incretin inactivation. The remaining three SNPs are located within a haplotype block towards the end of chromosome A3; within this region, genes of interest include *TMEM18* and *ACP1*, both previously associated with T2DM. This study indicates a polygenic component to susceptibility to DM in cats and has highlighted several loci and candidate genes worthy of further investigation.

## Introduction

Diabetes mellitus (DM) is a heterogeneous disease, characterised by a failure of glucose homeostasis, leading to persistent hyperglycaemia. In humans, the majority of affected individuals suffer from type 2 DM (T2DM), which is characterised by peripheral insulin resistance alongside defects in pancreatic beta cell secretion of insulin [1, 2]. Absolute or relative insulin deficiency leads to hyperglycaemia and abnormalities in carbohydrate, lipid and protein metabolism, as well as accelerated hepatic glycogenolysis and gluconeogenesis. T2DM is reaching epidemic proportions world-wide and represents a substantial proportion of the healthcare costs in many developed countries [3, 4]. Susceptibility to T2DM occurs as a consequence of a combination of environmental and lifestyle factors, mainly through obesity, inactivity, corticosteroid administration and secondary insulin resistance [5]; however, it is also known that T2DM has a strong genetic and epigenetic component [6, 7]. T2DM is a complex genetic disorder and several genetic studies, including genome-wide association studies (GWAS) have now identified more than 50 genes that are associated with the disease [8, 9]. Most of these susceptibility genes are associated with beta-cell biology, although the mechanisms through which they are linked to dysfunction and T2DM are still largely unknown.

DM is one of the most common feline endocrinopathies, with most cats suffering from a condition similar to T2DM in humans. The prevalence of DM in the overall cat population in the UK is of around 0.4%-0.58% (with an estimated cat population of 7.3 million) [10, 11], depending on the study, or 0.01% in the US with a 15 fold increase in a 30 year period [12] and 0.74% in Australia [13]. The disease is most commonly seen in domestic shorthair (DSH) and domestic longhair (DLH) cats [11, 14]. Other studies from Australia, the UK, USA and New Zealand have shown that mainly Burmese cats are overrepresented in the diabetic population [11–15]. Other breeds with increased odds ratio include Norwegian Forest, Tonkinese, Russian Blue and Oriental, whereas other pure breeds are underrepresented such as Bengal, Birman, Ragdoll, British Short Hair amongst others [11, 14]. The evidence of breed predisposition supports a genetic underlying mechanism for the development of T2DM in the cat.

Similar to the situation in humans, susceptibility to feline DM is thought to be influenced by environmental and genetic risk factors [10, 16–21]. Although little is known about the genetic risk factors in feline DM, human type 2 diabetes (T2DM) and feline DM share many of the environmental risk factors, such as obesity, physical inactivity, eating dry food and corticosteroid administration [10, 12, 22]. A polymorphism in the coding sequence of the melanocortin 4 receptor *(MC4R*) gene has been reported to be associated with feline DM, in a population of overweight DSH cats in the UK [21]. Recent studies in Burmese cats have also suggested the possibility that this breed has a genetically-conferred derangement of lipid metabolism, which might predispose them to developing DM, however, this study only evaluated non-diabetic cats and therefore the impact of this derangement in the diabetic Burmese population has not been determined [23].

Given the similarity in the pathophysiology, clinicopathological consequences and risk factors when comparing human T2DM and feline DM, it seems possible that feline DM could be a good model for the study of this disease in people [24] and it seems also possible that similar genetic and epigenetic susceptibility factors might be involved in development of the disease in both species.

Recently, the release of the feline genome assembly and availability of feline SNP (Single Nucleotide Polymorphism) genotyping arrays has allowed GWAS to be undertaken for breed-specific diseases such as hypokalaemia in Burmese cats [25] or breed susceptibility to feline infectious peritonitis [26], amongst others [27, 28]. For feline DM, two recent GWAS analysis in this breed identified several candidate genes [20], as well as genomic regions involved in lipid metabolism, insulin sensitivity and Beta-cell dysfunction as likely to be associated with diabetes mellitus in this breed [19]. The aim of the present study was to identify genetic factors associated with susceptibility to DM in non-obese DSH cats by using GWAS as a tool.

## Materials and methods

### Study population

Blood samples (EDTA or clots from serum tubes) from diabetic cats (n = 200) were recruited at the Queen Mother Hospital for Animals, Royal Veterinary College or from first opinion practices, through the UK Companion Animal Diabetes Register. External samples were submitted through a standardized submission form, which included information about signalment, presence of concurrent diseases, clinical signs, body condition score (BCS) at sample submission and at/before diagnosis, diet and insulin type and dose. BCS assessment was carried out according to published 9- and 5-point BCS systems [29, 30]. Diabetic cats were selected on the basis of the presence of persistent hyperglycaemia and elevated serum fructosamine concentration with all cats requiring insulin treatment. Insulin-like growth factor-1 (IGF-1) was measured in all samples to exclude hypersomatotropism-related DM. DSH cats with an IGF-1 concentration <800 ng/ml and BCS ≤ 4/5 or 5/9 and/or BW <4kg were included in the study.

Blood samples (EDTA or clots) from non-diabetic cats (n = 399) were obtained from the Royal Veterinary College sample archive, consisting of residual samples after completion of diagnostic testing from referral and first opinion cases seen at the Queen Mother Hospital for Animals and the Beaumont Sainsbury Animal Hospital. Only DSH cats that were 10 years old or older at the time of sample collection and with no clinical or biochemical evidence of DM were included. There were no known familial relationships between any of the cats included in the study. The Royal Veterinary College’s Ethics and Welfare Committee approval was granted for use of blood samples for clinical research (URN 2011–120).

### DNA extraction

Genomic DNA was extracted from blood samples using the GenElute Blood Genomic DNA Kit (Sigma-Aldrich, UK) according to the manufacturer’s instructions. The concentration of DNA was measured using a NanoDrop 1000 spectrophotometer (NanoDrop products, Wilmington, Delaware, USA). Genomic DNA samples were submitted to Geneseek Inc. (Lincoln, NE, USA) for SNP genotyping using the Illumina Infinium 63k iSelect DNA array (Illumina Inc., San Diego, USA).

### Genome-wide association study

#### Population structure and quality control

Quality control was performed for DNA from 599 cats (200 diabetic, 399 controls) and 62897 SNPs using PLINK 1.9 [31]. Possible duplicate samples were identified using the–*genome* operator, which looks at the genetic relatedness of all pairs of samples. Samples with PI_HAT > 0.6 were identified as duplicates and the individual in each duplicate pair with the highest level of SNP missingness was removed. Population structure was examined using QQ plots and Multi Dimensional Scaling (MDS) plots, using PLINK 1.9 and EMMAX [32]. Quality control of the SNP was performed by removing those with minor allele frequency below 0.05 (-*maf*), or a variant call rate (*-geno*) below 0.9. Samples with a genotyping rate (-*mind*) of less than 0.9 were also removed.

#### Case-control genome-wide association analysis

The genotyping data was analysed using PLINK 1.9 whole genome data analysis toolset. A map document (S1 Table) with the chromosomal location of each SNP aligned to the latest Feline assembly (FelCat9) was used for analysis [19]. The case-control association analysis was performed on the available samples (-assoc). The association results from PLINK were plotted as -logp values (p_raw_) and also as max(T) empirical p values (p_genome_) using the–*mperm 100*,*000* option in PLINK, to correct for multiple testing. Max(T) permuted p-values were considered significant at a p_genome_ value <0.05. Data for the Manhattan plots were created using PLINK. Once the analysis was finalised, those SNPs above the significance threshold were located within the latest feline genome assembly (Felis catus 9.0) to look for possible candidate genes.

#### Haplotype analysis

Haplotype analysis was carried out using HAPLOVIEW [33] to determine whether the significant SNPs were in linkage disequilibrium (LD) with any possible candidate genes. Using the–*clump* option of PLINK, with linkage disequilibrium set at r^2^ > 0.2, SNPs were also grouped based on LD. Significance of DM-associated haplotype blocks was measured by running 10,000 permutations in HAPLOVIEW.

## Results

### Study population

Genomic DNA from a total of 200 non-obese diabetic DSH cats and 399 non-diabetic DSH cats was submitted for genotyping. The mean (SD) age of the diabetic population was 11.62 (3.44) years, range 2–21 years. One hundred and twenty eight cats (64%) were male, three of them entire; seventy one cats (36%) were female (five were female entire), sex was unknown in one diabetic cat. The mean (SD) weight of the diabetics was 4.35 (1.01) kg. The mean (SD) age of the control cat population (n = 389) was 14.83 (2.06) years, range 11–24 years. One hundred and eighty two cats (46%) were female, one of them female entire and the rest were female neutered; two hundred and sixteen (54%) were male, neutering status was not known in one cat, the rest were neutered, sex was unknown in one cat. Of the control cats, two hundred and thirty nine (60%) had lean body condition score, seventy six (19%) had overweight body condition score and 84 (21%) had an unknown body condition score.

### GWAS

A total of 599 cats were analysed (200 cases, 399 controls). After excluding duplicated samples (n = 8, 3 cases, 5 controls), samples without sex information (n = 2, 1 case, 1 control) and samples with insufficient genotyping rate (n = 2, 1 case, 1 control), 195 diabetic cats and 392 controls were accepted for further analysis. SNPs that did not meet the genotyping (n = 1,462 SNPs) and frequency (n = 11,279 SNPs) criteria were also excluded from further analysis, there were 47,497 SNPs for analysis.

**Fig 1:**
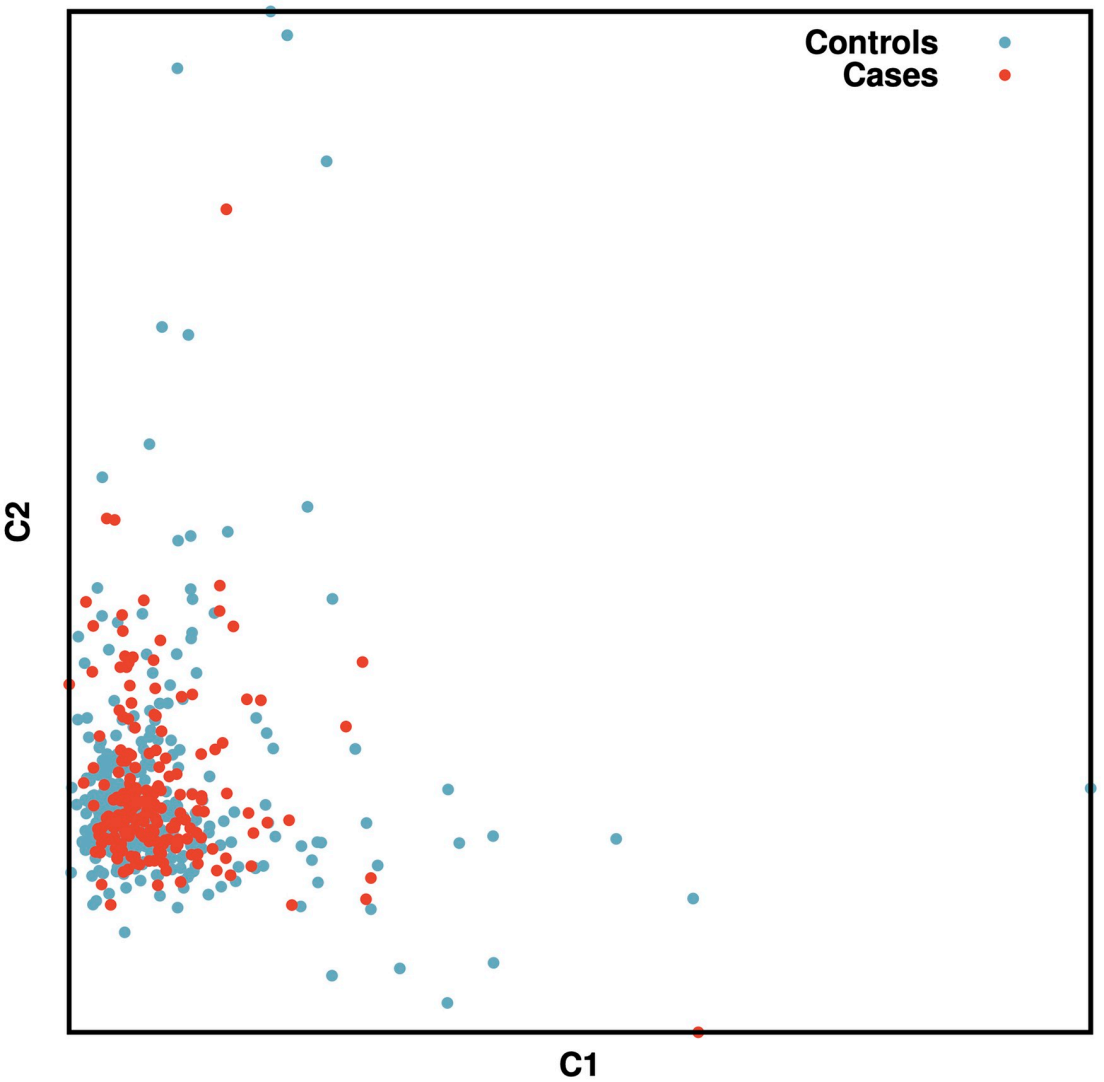
MDS plot of all cats included in the GWAS. On the X axis is the first dimension of the IBS calculation, the Y axis contains the second dimension. The blue dots represent the control cats, while the red dots represent the DM cases.

An MDS plot was performed to test for population stratification. This revealed that most cats were within one main cluster, and that both cases and controls seemed to be randomly distributed within the cluster (Fig 1).

**Fig 2:**
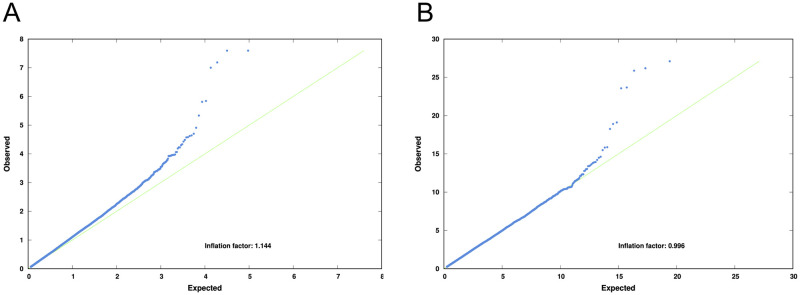
Quantile-quantile plots of Chi-Squared values before (A) and after (B) correction by EMMAX. The blue dots are the SNPs. On the Y axis, the observed Chi-Square value for each SNP, on the X axis is the expected value for each SNP. The green line represents when both expected and observed values coincide (inflation factor = 1).

A quantile-quantile (QQ) plot of the data revealed an inflation factor of 1.146, suggesting that there may be some relatedness or structure within the population. After the data was processed using linear mixed model association (EMMAX) the inflation factor was 0.995, demonstrating that EMMAX adjustment had largely coped with any population structure (Fig 2).

Four SNPs are significant genome-wide at the p<0.05 level after mperm correction (p_genome_), one on chromosome A2 and three on chromosome A3: chrA2.4150731 (p_genome_ = 0.039); chrUn17.115508 (p_genome_ = 0.022); chrUn17.394136 (p_genome_ = 0.016); chrUn17.314128 (p_genome_ = 0.016) (Table 1).

**Table 1:** Summary of results after association and haplotype analysis.

Chr	Locus	Type	Position	p_raw_	p_genome_	Alleles in locus	Minor allele	Case/contr freqs minor allele	Genes within haplotype block
A3	chrUn17.394136	SNP	142804428	2.53 x10^-8^	0.016	C/A	C	0.31, 0.48	*1*
									*TMEM1*
									*ACP-18*
A3	chrUn17.314128	SNP	142885267	2.53 x10^-8^	0.016	G/A	G	0.31. 0.48	*TMEM18*
									*ACP-1*
									*SHY3YL1*
A3	chrUn17.115508	SNP	143084579	6.51x10^-7^	0.022	A/G	A	0.31, 0.48	*TPO*
									*TMEM18AACP1*
									*SHY3YL1*
									*FAM110C*
A3	GAGAA	Haplotype block	142804428–143195425	5.13 x10^-8^	0.0002			0.30, 0.47	TMEM18
									ACP1
									SHY3YL1
									FAM110C
A2	chrA2.4150731	SNP	3535683	9.94 x10^-8^	0.039	A/C	A	0.31, 0.17	*DPP9*

The top most significant loci, with their positions in the FelCat9 assembly, as well as the frequencies in cases and controls, p_raw_ and p_genome_ values and genes in proximity (within 200kb of the SNP) are displayed. Also shown is the haplotype block identified in HAPLOVIEW, and the p-value after running a haplotype association test in Haploview, with and without permutations.

**Fig 3:**
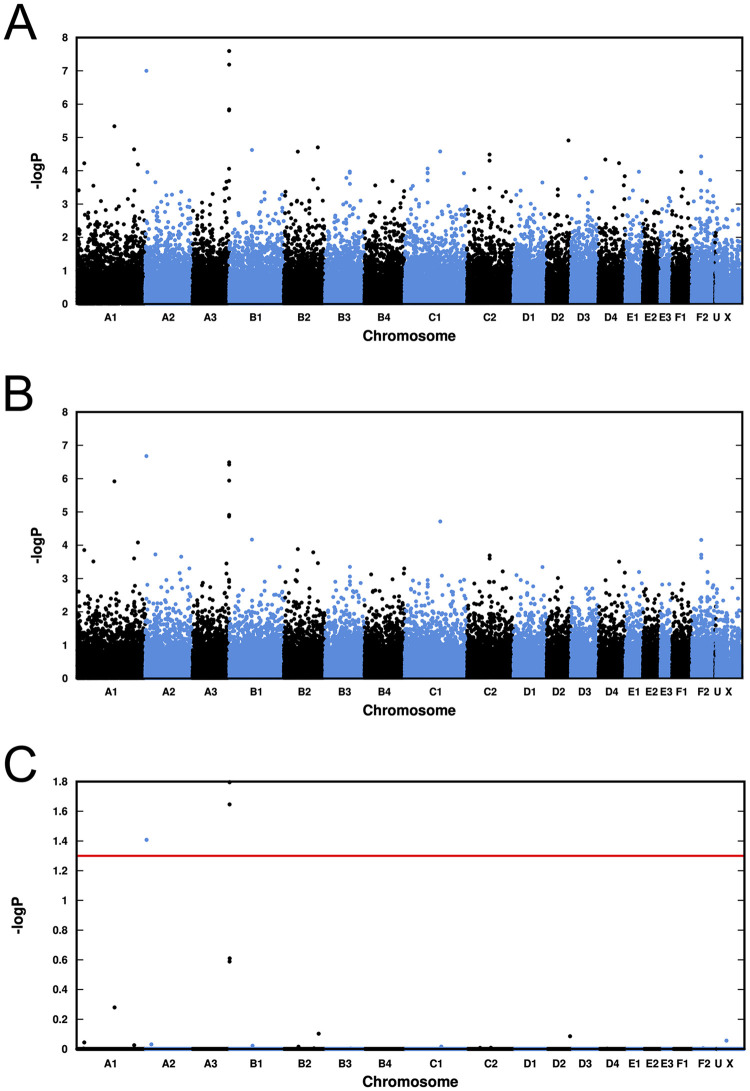
Association plots of our case-control genome-wide association analysis. A) Standard association plot of p_raw_ values. B) Plot of EMMAX-corrected association values. (C) Plot of p_genome_ values after correction for multiple testing by the mperm option of PLINK. The red horizontal line shows the p = 0.05 level of significance. In all plots the chromosome marked “U” contains SNPs which are mapped to the new assembly but not to one of the standard chromosomes.

Fig 3 shows a comparison of the association plots of the data before and after correction for population structure by EMMAX, and also a plot of the p_genome_ values calculated by PLINK mperm 100,000. The top SNP in chromosome A2 has almost maintained its pre-correction value. The “peak” of SNPs in chromosome A3 is also still clearly present, though at a slightly lower value.

### Haplotype analysis

**Fig 4:**
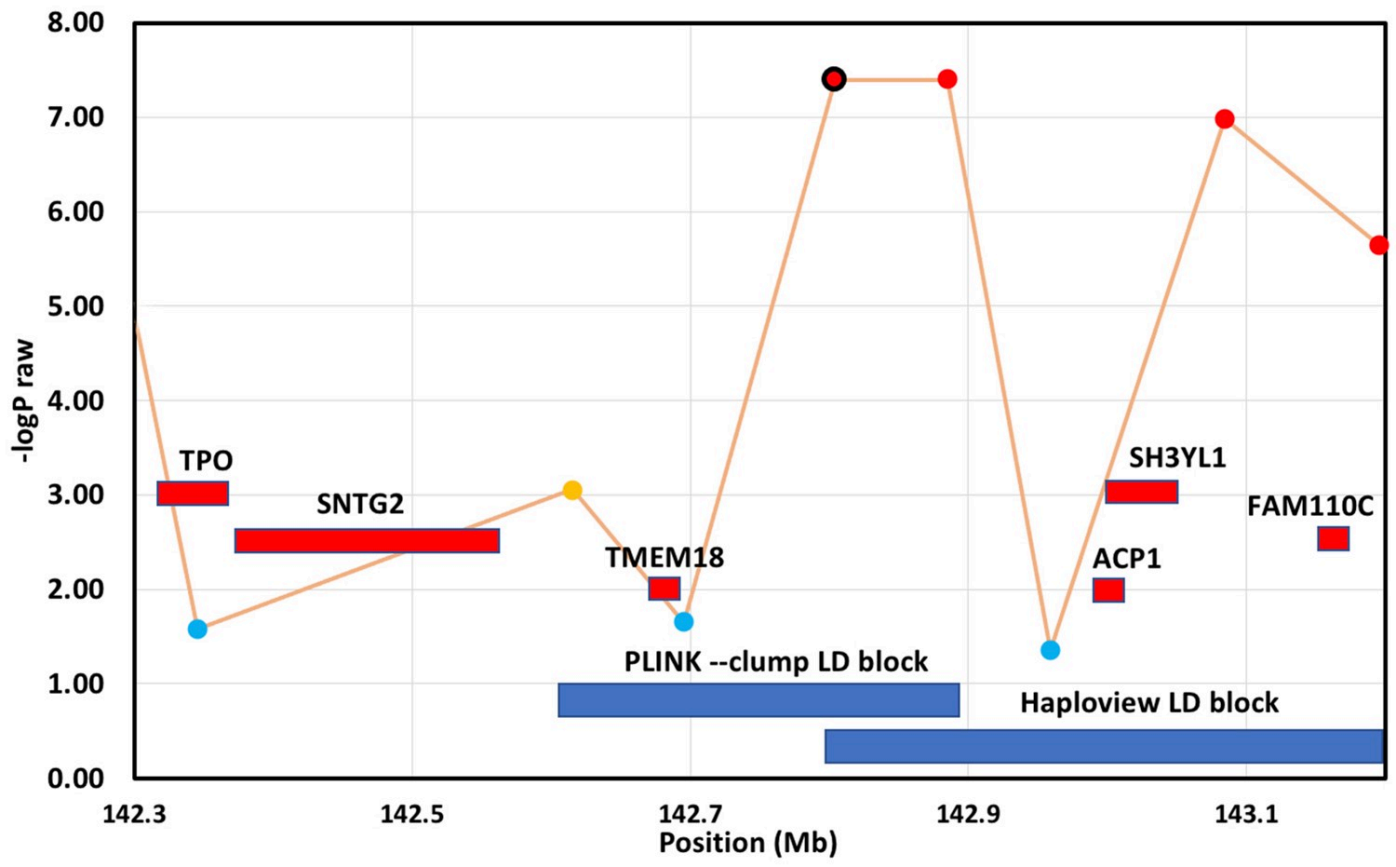
Haplotype blocks around the significant region on chromosome A3. The block identified by PLINK–clump, and the block identified by HAPLOVIEW are shown in blue. SNP variants are plotted as -log10 p_raw_, and are shown with colours indicating the amount of LD with the most significant SNP, as follows: red (r2 > 0.8), orange (r2 > 0.2) and blue (r2 < 0.2). The rightmost SNP is at the end of the chromosome.

A haplotype block enclosing the three top SNPs was identified (see Table 1 and Fig 4). This was located in chromosome A3 (p_raw_ 5.13 x10^-8^, p_genome_ 0.0002) and is present in 30% of the cases and 47% of the controls.

The significant SNP (chrA2.4150731) on chromosome A2 was not part of any haplotype detected, but it does lie within 4kb of the start of the *DPP9* gene, so is likely to act as a good proxy marker for the gene.

PLINK–*clump* and HAPLOVIEW identify slightly different haplotype blocks, but both are overlapping with the top SNP. The two closest genes in this area of LD are *TMEM18* and *ACP1*.

## Discussion

This study aimed to identify novel loci associated with Feline DM in a population of lean DSH cats in the United Kingdom. The Illumina 63k feline SNP array was used for this purpose. In this GWAS, a group of 195 lean DSH diabetic cats was compared with 392 DSH controls. In this type of study, where phenotypic classification of cases and controls is of high importance [34], it is possible that selection of cats could have influenced the results. Within the DSH population, cases and controls were identified on the basis of their electronic patient record or information provided on sample submission forms by first opinion veterinary practitioners. Although careful selection took place, according to the information available, the authors did not have full access to the patient’s medical records. With regards to the control cats, the main limitation is that DM is a disease of late onset and therefore some of the control cats chosen for this study might have developed diabetes later in life. In order to minimise this, an attempt was made to select cats as old as possible, and with a diagnosis as robust as possible. All control samples were selected from a population of relatively geriatric cats with a multitude of diseases and this could also have influenced the results. The diabetic population in this study was chosen to have lean body condition. Records of body weight in the control population were not consistently present, but body condition score was available for a majority of them and 60% of control cats were found to be lean, which makes it unlikely that this study could have detected markers for lean body condition as well as for diabetes.

The SNP arrays used for these GWAS were the first manufactured for the feline species. However, the overall number of SNPs is somewhat limited, when compared to the ones used in human studies and other veterinary species such as dogs [35, 36]. This could have influenced the results [34], as it is possible that regions of interest might not have sufficient coverage within this array and therefore some potentially important gene associations could have been missed. Although the relatively low number and quality of SNPs in the array could have influenced the results, several GWAS have been published using the same SNP array for the research of feline diabetes [19, 20], albeit in a more inbred population such as the Burmese cats. Some of the limitations of the current SNP arrays have been overcome by performing haplotype analysis to further limit the presence of type 1 errors and to allow genes of interest that are in linkage disequilibrum (LD) to be determined.

Population structure was evaluated by MDS plots generated for each analysis. The initial MDS plot showed a large cluster with both cases and controls randomly distributed within it. Although the DSH is considered an outbred population, the genotyping data was also assessed using EMMAX, to investigate the effect of hidden relatedness and population stratification on the results. EMMAX, uses a linear model approach (LMM) and is currently preferred to traditional family-based association tests [32]. Quality control criteria for genotyping rate and testing for duplicates was also performed prior to the association analysis.

This GWAS has identified several SNPs that have reached genome-wide significance: 3 in the same region of Chromosome A3: A3:142,804,428, (p_genome_: 0.016); A3:142,885,267 (p_genome_: 0.016) and A3:143,084,579 (p_genome_: 0.022). All three are included in a haplotype block that includes genes linked to human T2DM, including *TMEM18* [37–39] and *ACP1* [40, 41]. Although not much information is available about the exact role that these genes play in T2DM in humans, they have been reported to be associated with the disease in different populations. In the case of *TMEM18*, it has been linked to obesity in multiple studies, which would seem an interesting contrast with the results of this study, as SNPs near this gene were identified in our cohort of non-obese diabetic cats. However, more recent studies have reported that the association of TMEM18 with T2DM in humans is independent of BMI [38, 42].

Also in this region of chromosome A3 is the *ACP1* gene, encoding a phosphatase that is thought to play a role in regulating glycolytic rate through control of insulin receptor activities [40]. *ACP1* polymorphisms have been linked to insulin resistance. The increased risk of T2DM associated with *ACP1* seems to be mediated through increased risk of obesity in humans [41], although one study found an association with a polymorphism in this gene with insulin resistance in males, irrespective of BMI [43]. It is possible that these two genes are not associated with feline DM and other genes within this region of chromosome A3 are involved (Table 1), however, given their established link with T2DM in humans, *TMEM18* and *ACP1* should be assessed more closely in future investigations.

The significant SNP in chromosome A2 (A2: 3535683; p_genome_ 0.039) was not part of any haplotype block detected, but it does lie within 4kb of the start of the *DPP9* gene, so is likely to be inherited with mutations in this gene. *DPP9* belongs to a family of serine-proteases, the dipeptidyl-peptidases, and is highly similar in sequence to dipeptidyl-peptidase 4 (*DPP4*). Although their specific function is not fully described, it has been shown to play a role in post-prandrial insulin secretion as well as in adipogenesis [44, 45]. *DPP4* inhibitors are currently being used as part of the medical management of T2DM in humans, as they improve glycaemic control through increasing GLP-1 concentrations, thus stimulating pancreatic insulin secretion, inhibiting glucagon release and reducing appetite [46]. Many of these *DPP4* inhibitors also have an inhibitory effect on DPP8 and *DPP9* [47], which is again suggestive of similar biological function. Interestingly, *DPP4* inhibitors have been investigated recently for the management of DM in cats, with somewhat less success than in humans [48–50]. It would therefore be interesting to further investigate the role of the *DPP9* gene in feline DM and whether this could be linked to the relative lack of success obtained with these drugs, when compared to humans with T2DM. Up until now, no variants of *DPP9* have been linked to T2DM, but it is possible that this study could lead to the identification of novel genetic associations for T2DM and feline DM.

The lack of a haplotype block around the *DPP9* gene may be affected by the fact that the density of SNPs in the array is relatively low in this specific region: the SNP upstream of the current significant SNP is 83kb away, and the next SNP downstream is 38kb away. When comparing this with the SNP density in the region of chromosome A3, for the most significant SNPs near the *ACP1* and *TMEM18* genes, the upstream SNP is 18kb away and the next downstream SNP is 8kb away, showing that the SNP density is higher in the region of interest on chromosome A3 than in our region of interest in chromosome A2. The DSH breed used in this study is considered to be relatively outbred, which implies shorter haplotype blocks. Although no current studies have specifically looked at the degree of LD present in the DSH population in the UK, a recent study looked into the degree of LD in several cat breeds, including a random bred population [51]. The extent of LD in the random bred population was of approximately 18Kb. This fact, together with the sparcity of SNPs in this region could also explain why no haplotype blocks were identified in Chromosome A2.

## Conclusions

Several SNPs have been identified which provide moderate to strong evidence of association to DM in the cat being located within a haplotype block spanning genes that have been associated with human diabetes (*ACP1* and *TMEM18*). An independent significant SNP has also been identified within 4Kb of a gene that could have a pathophysiological link with DM, based on its function (such as *DPP9*). Further investigations could involve more detailed investigation of the regions and genes of interest highlighted in this GWAS. Additionally, further genetic analysis, preferably in an independent population and making use of higher density SNP genotyping arrays to assess for repeatability of these findings might help to confirm the associations identified in the present study and to detect further associations.

## Supporting information

S1 Table Updated map file according to FelCat9 assembly. (XLSX)
